# Psychiatric Symptoms, Posttraumatic Growth, and Life Satisfaction Among Parents of Seriously Ill Infants: A Prospective Case-Controlled Study

**DOI:** 10.1007/s10880-022-09868-7

**Published:** 2022-03-28

**Authors:** Krista Koivula, Siiri Isokääntä, Kati Tavast, Iines Toivonen, Iina Tuomainen, Merja Kokki, Kirsi Honkalampi, Ulla Sankilampi, Hannu Kokki

**Affiliations:** 1 Department of Pediatrics, Kuopio University Hospital (KYS), Puijonlaaksontie 2, PO Box 100, 70029 Kuopio, Finland; 2 Department of Anesthesiology and Intensive Care, Kuopio University Hospital, Kuopio, Finland; 3 Present Address: School of Medicine, Faculty of Health Sciences, University of Eastern Finland, Kuopio, Finland; 4 School of Educational Sciences and Psychology, University of Eastern Finland, Joensuu, Finland

**Keywords:** Anomaly, Asphyxia, Infant, Parent, Psychiatric symptoms, Resilience

## Abstract

We evaluated psychiatric symptoms, posttraumatic growth, and life satisfaction among the parents (*n* = 34) of newborns (*n* = 17) requiring therapeutic hypothermia or urgent surgery (interest group). Our control group included 60 parents of healthy newborns (*n* = 30). The first surveys were completed soon after diagnosis or delivery and the follow-up surveys 1 year later (participation rate 88% in the interest group and 70% in the control group). General stress was common in both groups but was more prevalent in the interest group as were depressive symptoms, too. Anxiety was more common in the interest group, although it showed a decrease from the baseline in both groups. Life satisfaction had an inverse correlation with all measures of psychiatric symptoms, and it was lower in the interest group in the early stage, but similar at 12 months due to the slight decline in the control group. Mothers in the interest group had more anxiety and depressive symptoms than fathers in the early stage. Mothers had more traumatic distress than fathers at both time points. Half of the parents experienced substantial posttraumatic growth at 12 months. In conclusion, the serious illness of an infant substantially affects the well-being of the parents in the early stages of illness and one year after the illness.

Having and caring for a seriously ill infant in a neonatal intensive care unit (NICU) is a heavy burden for parents and affects their psychological well-being in several ways. The uncertainty associated with child’s illness and survival, intense treatments, and the prolonged hospital stay cause distress and concern. Parents may experience posttraumatic stress and trauma symptoms, as well as anxiety, depression, and general stress (e.g. Cabizuca et al., 2009; Muscara et al., 2015; Yaman & Altay, 2015).

In previous studies, half of the parents of children with a life-threatening illness treated in cardiology, oncology, and pediatric intensive care units have met the criteria for acute stress disorder (Muscara et al., 2015). Fathers appear to experience the same level of psychological distress and psychiatric symptoms as mothers when their child is hospitalized in a pediatric intensive care unit (Khoddam et al., 2021). Despite research on stress in the parents of infants in intensive care, data on the parents of infants suffering from birth asphyxia and severe congenital anomalies are limited, and little attention has been paid to posttraumatic growth (PTG) and life satisfaction (LS) in these families. Moreover, previous research has paid less attention to the symptoms in fathers than in mothers, even though fathers are closely involved in the care of their newborn infant in current family centered wards with single family rooms. The inclusion of both parents in research is justified, because recent studies have demonstrated that fathers also have clinically important anxiety and risk factors for posttraumatic stress disorder (PTSD), and their coping strategies differ compared to mothers (Aftyka et al., 2017a, 2017b; Dhingra, 2020; Khoddam et al., 2021). In the study of Aftyka et al. (2017a, 2017b), PTSD was present in 60% of the mothers and 47% of the fathers of infants who were hospitalized in the NICU during the neonatal period, and the mothers had higher levels of stress and a higher severity of PTSD than the fathers. Despite having less stress, fathers often experienced a sense of lack of control and helplessness when they had an extremely ill infant in intensive care (Arockiasamy et al., 2008; Khoddam et al., 2021).

Parents whose child is experiencing pediatric medical traumatic stress caused by pain, injury, serious illness, medical procedures, and invasive or frightening treatment experiences, have also shown resilience (e.g. Isokääntä et al., 2019) and PTG (e.g. Hungerbuehler et al., 2011). PTG appears to be more pronounced in mothers than fathers (Aftyka et al., 2020). Resilience, in the broadest sense, refers to dynamic processes that lead to adaptive outcomes in the face of adversity, and PTG is defined as “positive psychological change experienced as a result of a struggle with highly challenging life circumstances” (Tedeschi & Calhoun, 2004). Components of PTG may include improved interpersonal relating, a greater appreciation for life, a sense of personal strength, new life possibilities, and spiritual change (Park, 2017; Tedeschi & Calhoun, 1996). In order to develop appropriate interventions, it is important to understand the factors that affect parental PTG, distress, and anxiety, and data on both parents are needed. It is known that PTSD in fathers is related to that in their partners (Lefkowitz et al., 2010). Higher anxiety scores of parents have been associated with the use of self-blame, lower optimism scores, higher levels of illness-related uncertainty, and a greater number of previous hospital stays (Wray et al., 2011). Shame and fear of death have substantial actor effects on distress in both mothers and fathers, whereas chronic guilt has a stronger effect on maternal distress (Barr, 2015). Pregnancy and childbirth are intensive emotional and physical experiences for the mother, which may also contribute to explaining the differences in parental experiences.

The psychiatric symptoms of parents strongly influence their own well-being, the parent–child relationship, and, through interaction and care, also the well-being, development, and behavior of the child. Posttraumatic stress symptoms in parents following their child’s severe diagnosis or hospital admission predict higher healthcare service utilization in the following 12 months (Thompson et al., 2017). The psychological well-being of parents is also crucial for the treatment of a child’s illness. Posttraumatic arousal and re-experiencing symptoms may impair the ability of parents to comprehend medical guidelines and communicate essential information about the child’s well-being to healthcare professionals (Kazak et al., 2004). On the one hand, hypervigilant parents may request extra doctor visits or have frequent contacts with medical services, consequently overburdening the healthcare system and leading to additional costs (Pelcovitz et al., 1998). On the other hand, posttraumatic avoidance symptoms can cause parents to avoid important medical visits and procedures (Stuber et al., 1996). Arousal symptoms may induce parents to overprotect their children and restrict their participation in activities, thereby hindering their normal development (Santacroce, 2002). Traumatic stress disorder is associated with an increased risk of depression and substance abuse, which may also impair the ability of parents to respond to the child’s developmental needs (Cabizuca et al., 2009).

Birth asphyxia and its treatment is an example of a medical condition that can cause substantial traumatic stress in parents (de Haan et al., 2006). Birth asphyxia with hypoxic-ischemic encephalopathy is treated with therapeutic hypothermia, and manipulation is kept minimal. Thus, it is not possible for parents to hold the newborn during the first 72 h (Long & Brandon, 2007). These specific circumstances may affect the experience of becoming a parent and the establishment of the parent–infant relationship (Heringhaus et al., 2013). Newborns may also need surgical or other invasive procedures and admission to the NICU soon after birth. Some congenital anomalies, such as omphalocele, gastroschisis, and esophageal atresia, require surgical treatment during the first days after birth (Gamba & Midrio, 2014; van der Zee et al., 2017). Taken together, parents of an infant admitted to the NICU during the first days after birth are concerned about their newborn infant’s current and future health, well-being, and survival. During the child’s recovery, some parents develop heightened parental perceptions of child vulnerability, which leads to a pattern of overprotective parenting and may result in adverse neurodevelopmental and behavioral outcomes in the child over time (De Ocampo et al., 2003; Hoge et al., 2021).

Parents use several strategies for adapting to these challenging situations, and nurses play a pivotal role in providing support for parents in the NICU (Nassef et al., 2013). Hall’s study (2005) implies that healthcare professionals need to help parents understand events related to the treatment of their infant and to instill hope, despite the unknown outcome. Healthcare professionals should also accept and respect the parents’ coping strategies for stress and their concerns. Hence, it is important for healthcare professionals to know and recognize the impact of the illness and the treatment on the psychological well-being of the parents to maximize the quality of family centered care in hospital.

In this study, we compared symptomology across the parents of ill and healthy infants and between fathers and mothers. Research on the psychological well-being of both parents of critically ill infants helps healthcare professionals to understand parents’ reactions, identify parents in need of professional help, and guide them to obtain appropriate support, treatment, and counselling. Identification of the psychological effects and counselling of parents to obtain the required help will improve the quality of healthcare services and facilitate the treatment of the infant’s illness. Responding to the need for parental support may also prevent subsequent mental health problems in children and parents. The aim of this study was to examine the prevalence of psychiatric symptoms, PTG and LS among these parents.

## Methods

### Participants

This study included two cohorts: parents of infants (*n* = 17) with a serious illness, forming the interest group (*n* = 34; 17 dyads), and parents of healthy infants (*n* = 30), forming the control group (*n* = 60; 30 dyads). The parents were recruited from Kuopio University Hospital, Kuopio, Finland, between April 2014 and April 2018. In the interest group, the parents of 24 infants were invited to participate and 17 couples agreed, while in the control group, 30 couples of the 34 who were invited agreed to participate.

Family characteristics are listed in Table 1. The participants were all heterosexual couples, and the age distribution of the participants was similar in both groups. The cohorts had similar rates of previous miscarriage, but the interest group had slightly higher rates of infertility treatment (24% vs. 10%). Paternal employment rates were similar in both groups, but in the interest group, only half of the mothers were employed compared to 80% in the control group. Furthermore, the level of education was higher in the control group than in the interest group. Seven of the 94 parents had a background in healthcare; there were three nurses in the interest group and three physicians and one nurse in the control group. All except one couple in the interest group were first-time NICU parents. Economic (unemployment/low incomes/debts/distraints) and other burdens in the family (stress at work, divorce, lack of social support, moving to a new house, selling a house) were also more common in the interest group. Physical and mental illness rates were relatively similar, but physical illnesses in mothers and mental illnesses in fathers were slightly more common in the control group, and mental illnesses in mothers in the interest group.

**Table 1:** Background data of the families (*N* = 17 dyads/34 parents in the interest group and 30 dyads/60 parents in the control group)

Variable	Interest group *(N* = 34)		Control group (*N* = 60)	
Age (years)				
Fathers	*n* = 17	30 [21–47]	*n* = 30	31 [21–47]
Mothers	*n* = 17	28 [21–39]	*n* = 30	29 [20–38]
Miscarriages *n* (%)	4 (24%)		7 (23%)	
Infertility treatments *n* (%)	4 (24%)		3 (10%)	
Mode of delivery *n* (%)				
Vaginal	9 (53%)		29 (97%)	
Cesarean	8 (47%)		1 (3%)	
Length of NICU stay days	12 [3–37]		-	
Mothers employed *n* (%)	8 (47%)		24 (80%)	
Fathers employed *n* (%)	16 (94%)		27 (90%)	
Education *n* (%)				
Basic level	1 (3%)		1 (2%)	
Upper secondary	16 (47%)		25 (42%)	
Lower-degree tertiary	11 (32%)		12 (20%)	
Higher-degree level tertiary	1 (3%)		11 (18%)	
NA	5 (15%)		11 (18%)	
Burden in family				
Economic *n* (%)	7 (41%)		5 (17%)	
Other *n* (%)	4 (24%)		3 (10%)	
Physical illnesses				
Fathers *n* (%)	4 (24%)		8 (27%)	
Mothers *n* (%)	2 (12%)		6 (20%)	
Mental illnesses				
Fathers *n* (%)	1 (6%)		4 (13%)	
Mothers *n* (%)	5 (29%)		7 (23%)	
Siblings in the family (yes/no)	9/8		11/19	
Number of siblings	1 (0–7)		0 (0–6)	
Illnesses of siblings				
Physical *n* (%)	1 (5.9)		NA	
Mental *n* (%)	0 (0)		NA	

Data are median [range] or number of cases (%)

*NICU* neonatal intensive care unit

At recruitment, the infants were between 0 and 19 days old, and half of them had siblings, 53% in the interest group and 37% in the control group. Only a few of the siblings had illnesses. Infants in the interest group had a life-threatening illness or condition necessitating therapeutic hypothermia or urgent surgery and were admitted to the NICU for a median of 12 days; the diagnoses were asphyxia and hypoxic-ischemic encephalopathy (*n* = 9), gastroschisis (*n* = 3), esophageal atresia (*n* = 2), omphalocele (*n* = 2), and renal failure (*n* = 1). All infants in the control group were healthy and there was no need for NICU admissions.

### Procedure

Approval for the study protocol was obtained from the Research Ethics Committee of the Northern Savo Hospital District, Kuopio, Finland (No. 88/2013; January 7, 2014). The study had institutional approval (No. TJ_146/2015) and complied with the American Psychological Association Ethical Principles and the Ethical Principles presented in the Helsinki Declaration regarding the treatment of participants. The participants in the interest group were recruited by the first author (KK) in the NICU between April 2014 and April 2018 and those in the control group (HK, IT) in the labor ward of the hospital in May 2015. The inclusion criteria in the interest group were a life-threatening illness diagnosed in a full-term newborn and requiring urgent invasive treatment, either therapeutic hypothermia or surgery, a couple relationship with the child's other parent, and voluntary written consent from both parents. The inclusion criteria in the control group were a couple with a healthy, full-term biological newborn and that both parents consented to participate.

Participants were given oral and written information about the study and time to consider participation. The participants provided written informed consent. Refusal to participate in the study did not affect the treatment of an infant or parents in the hospital. Parents also had the right to withdraw their participation in the study at any time and without reason. The participants completed the first questionnaires during the first days after the infant's diagnosis or delivery (Time 1). An exception to this was the first Impact of Event Scale–Revised questionnaire (IES-R; Weiss & Marmar, 1997), which was completed by the parents in the interest group one month after the infant’s diagnosis or delivery. Parents in the interest group completed the second questionnaires at a scheduled in-person visit in the hospital at 12 months, and parents in the control group returned mailed questionnaires in a prepaid envelope at 12 months after the birth of their infant (Time 2). Only the parents in the interest group filled in the IES-R and the Posttraumatic Growth Inventory (PTGI; Tedeschi & Calhoun, 1996).

Seventy-two participants (77%) were reached for the follow-up at 12 months; the interest group had a participation rate of 88% (*n* = 30) and the control group 70% (*n* = 42). The missing data were due to the fact that the subjects declined participation (*n* = 18 in the control group), were not reached (*n* = 2), or they had moved away (*n* = 2). In the interest group, there were no cases of attrition, but one couple in the control group had divorced during the 12-month follow-up period.

### Measures

In addition to demographic variables (Table 1), the participants filled out the seven following measures (five of these in both groups, and the IES-R and PTGI in the interest group only). Total scores from all the measures were calculated and compared between groups, and between the two time points within the groups. Dichotomized scores were also calculated for all measures.

#### Anxiety

The Beck Anxiety Inventory (BAI; Beck et al., 1988) was used to assess the subjective anxiety of the participants. The BAI is a 21-item self-report measure. The items were rated on a four-point scale of increasing severity. The BAI was dichotomized to no or mild anxiety (BAI score 0–15) and moderate to severe anxiety (BAI score 16–63). The internal consistency with the current sample was very high at both time points (Cronbach’s alpha = 0.961).

#### Depressive Symptoms

The Beck Depression Inventory—Second Edition (BDI-II; Beck et al., 1996) and the Edinburgh Postnatal Depression Scale (EPDS; Cox et al., 1987) were used to assess the current depressive symptoms of the participants. The BDI-II is a 21-item self-report measure. The items were rated on a four-point scale of increasing severity. The BDI-II was dichotomized to no depressive symptoms (BDI-II score 0–13) and depressive symptoms (BDI-II score 14–63). Internal consistency with the current sample was very high at both time points (Cronbach’s alpha = 0.911). The EPDS is a 10-item self-report measure. The items were rated on a four-point scale of increasing severity. The EPDS was dichotomized to no depression (EPDS score 0–9) and possible depression (EPDS score 10–30). Internal consistency with the current sample was also very high at both time points (Cronbach’s alpha = 0.905).

#### General Stress

The Perceived Stress Scale (PSS-14; Cohen et al., 1983) was used to assess subjective experiences of psychological stress. The PSS-14 is a 14-item self-report measure. The items were rated on a five-point scale of increasing severity, and seven positively stated items were reversed to obtain scores for summing. The PSS-14 score was dichotomized to low stress (PSS-14 score 0–18) and moderate or high stress (PSS-14 score 19–56) (Cohen et al., 1983). Internal consistency with the current sample was very high at both time points (Cronbach’s alpha = 0.861).

#### Posttraumatic Growth

The Posttraumatic Growth Inventory (Tedeschi & Calhoun, 1996) was used to assess positive change as a result of the struggle with stressful experiences that the participants identified. The PTGI is a 21-item self-report measure. The items were rated on a six-point scale of increasing growth. The PTGI score was dichotomized to no meaningful positive change (PTGI score 0–41) and at least a small positive change (PTGI score 42–105) (Sawyer et al., 2012). Posttraumatic growth was measured at 12 months in the interest group. Internal consistency with the current sample was very high (Cronbach’s alpha = 0.934).

#### Traumatic Distress

The Impact of Event Scale–Revised questionnaire (Weiss & Marmar, 1997) was used to assess subjective distress caused by a traumatic event. The IES-R is a 22-item self-report measure. The items were rated according to how distressing each symptom was over the past seven days on a four-point scale of increasing severity. The traumatic distress score was dichotomized to PTSD unlikely (IES-R score 0–24) and PTSD likely (IES-R score 25–66). Internal consistency with the current sample was very high at both time points (Cronbach’s alpha = 0.915).

#### Life Satisfaction

The 4-item Life Satisfaction Scale (Koivumaa-Honkanen et al., 2000) was used to assess subjective interest and happiness in life, ease of living, and the loneliness of the participants. The LS-4 is a four-item self-report measure. The items were rated on a five-point scale of decreasing satisfaction. The LS-4 score was dichotomized to satisfied (LS-4 score 4–11) and dissatisfied (LS-4 score 12–20) (Koivumaa-Honkanen et al., 2000). Internal consistency with the current sample was high at both time points (Cronbach’s alpha = 0.716).

### Statistics

The data were recorded and analyzed using SPSS software (IBM SPSS Statistics 25, International Business Machines Corporation, Armonk, NY, USA). The normal distribution of continuous data was tested with the Kolmogorov–Smirnov test, the Shapiro–Wilk test, and visually from histograms. The internal consistency of each questionnaire was tested with Cronbach’s alpha. Normally distributed quantitative data were analyzed with two-sided unpaired and paired *t*-tests assuming equal variances. Non-normally distributed data were compared with the Mann–Whitney *U*-test. We used the Wilcoxon signed-rank test for dependent data, and the Chi-squared test, Fisher’s exact test, and McNemar’s test for categorical data. Spearman's rank correlation test was used to test for correlations between variables. The data are displayed as the number of cases and median (range), unless otherwise specified. *p*-values of ≤ 0.05 were considered statistically significant.

## Results

Sociodemographic and other characteristics of families after the birth of the infant (*N* = 47 dyads) are listed in Table 1. The medians (minimum, maximum) of the survey scores are listed in Table 2 and categorized data in Table 3. The dichotomized data of the two groups and at two time points are presented below, along with correlations between the LS-4 score and other measures.

**Table 2:** Median [range] scores of surveys in the two cohorts after birth (T1) and after 12 months (T2)

Variable	Anxiety (BAI), scale 0–63	Depression (BDI-II), scale 0–63	Depression (EPDS), scale 0–30	Perceived stress (PSS-14), scale 0–40	Life Satisfaction (LS-4), scale 4–20	Posttraumatic Growth (PTGI), scale 0–105	Posttraumatic Stress Disorder Symptoms (IES-R^7^), scale 0–66
Interest group at T1 (*N* = 34)							
All	6 [0–42]	4 [0–22]	7 [0–21]	18 [7–40]	7 [5–16]	–	13 [1–41]
Mothers	15 [1–42]	8 [2–22]	12 [2–21]	23 [7–40]	8 [5–14]	–	18 [6–41]
Fathers	5 [0–25]	2 [0–18]	4 [0–18]	16 [8–31]	7 [5–12]	–	9 [1–30]
Control group at T1 (*N* = 60)							
All	5 [0–26]	2 [0–19]	3 [0–13]	16 [4–33]	5 [4–9]	–	–
Mothers	5 [0–26]	3 [0–11]	4 [0–13]	17 [4–33]	5 [4–9]	–	–
Fathers	3 [0–19]	1 [0–19]	2 [0–10]	16 [6–28]	5 [4–9]	–	–
* P*-value between the groups at T1	0.048	0.019	< 0.001	0.006	< 0.001	NA	NA
Interest group at T2 (*N* = 30)							
All	5 [0–28]	8 [0–32]	7 [0–19]	20 [9–34]	7 [4–18]	42 [8–89]	9 [1–47]
Mothers	7 [1–27]	8 [2–32]	12 [2–21]	21 [9–34]	8 [5–13]	45 [19–89]	18 [1–47]
Fathers	2 [0–28]	7 [0–22]	4 [0–16]	19 [11–32]	7 [4–18]	41 [8–65]	5 [1–30]
* P*-value T1 vs. T2 in the interest group	0.018	0.265	0.242	0.874	0.411	NA	0.008
Control group at T2 (*N* = 42)							
All	3 [0–15]	4 [0–21]	3 [0–15]	18 [3–34]	6 [4–12]	–	–
Mothers	3 [0–15]	7 [0–21]	5 [0–14]	18 [3–34]	6 [4–12]	–	–
Fathers	3 [0–13]	2 [0–14]	3 [0–15]	18 [12–27]	6 [4–10]	–	–
* P*-value T1 vs. T2 in the Control group	0.005	0.002	0.094	< 0.001	< 0.001	NA	NA
* P*-value between the groups at T2	0.029	0.021	0.026	0.093	0.109	NA	NA

*BAI* Beck Anxiety Inventory, *BDI-II* Beck Depression Inventory II, *EPDS* Edinburgh Postnatal Depression Scale, *PSS-14* Perceived Stress Scale-14, *LS-4* Life Satisfaction Scale-4, *PTGI* Posttraumatic Growth Inventory, *IES-R* Impact of Event Scale-Revised

**Table 3:** Classification of different questionnaire scores after the birth (T1) and after 12 months (T2). Data are number of cases

Variable	Interest group at T1 (*N* = 34)	Interest group at T2 (*N* = 30)	Control group at T1 (*N* = 60)	Control group at T2 (*N* = 42)
Anxiety (BAI)^a^	19/5/8/2	20/8/0/2	44/9/6/1	36/4/0/0
Mothers, *n*	7/2/6/2	8/6/0/1	20/4/5/1	19/2/0/0
Fathers, *n*	12/3/2/0	12/2/0/1	24/5/1/0	17/2/0/0
Depression (BDI-II)^b^	27/5/2/0	25/3/1/1	59/1/0/0	36/4/1/0
Mothers, *n*	12/3/2/0	12/2/0/1	30/0/0/0	17/3/1/0
Fathers, *n*	15/2/0/0	13/1/1/0	29/1/0/0	19/1/0/0
Depression (EPDS)^c^	22/6/6	23/4/3	56/4/0	37/1/2
Mothers, *n*	7/6/4	11/2/2	28/2/0	19/1/1
Fathers, *n*	15/0/2	12/2/1	28/2/0	18/0/1
Perceived stress (PSS-14)^d^	16/18/ 0	10/20/0	40/20/0	24/18/0
Mothers, *n*	5/12/0	4/11/0	18/12/0	11/10/0
Fathers, *n*	11/6/0	6/9/0	22/8/0	13/8/0
Life satisfaction (LS-4)^e^	12/20/2	9/15/6	47/13/0	21/19/2
Mothers, *n*	4/12/1	2/9/4	23/7/0	10/9/2
Fathers, *n*	8/8/1	7/6/2	24/6/0	11/10/0
Posttraumatic growth inventory (PTGI)^f^	NA	15 /15	NA	NA
Mothers, *n*	NA	8/7	NA	NA
Fathers, *n*		7/8		
Posttraumatic stress disorder symptoms (IES-R)^g^	20/3/3/6	21/3/2/4	NA	NA
Mothers, *n*	9/1/1/5	8/3/1/3	NA	NA
Fathers, *n*	11/2/2/1	13/0/1/1	NA	NA

^a^No/mild/moderate/severe anxiety

^b^No/mild/moderate/severe depression symptoms

^c^No depression/depression/severe depression

^d^Low/moderate/high stress

^e^Satisfied/slightly dissatisfied/dissatisfied with life

^f^At least a small positive change/no meaningful positive change

^g^No PTSD symptoms/some PTSD symptoms/PTSD/severe PTSD

### Anxiety

Early after the birth of the infant (Time 1), anxiety (BAI score ≥ 16/63) was more common in the interest group than in the control group (*p* = 0.032). Anxiety was more common among mothers than fathers in the interest group (*p* = 0.024), and similar in both sexes in the control group (*p* = 0.103) (Table 3).

Twelve months later (Time 2), anxiety was less common. Two out of 30 parents in the interest group, one mother and one father, had a BAI score of ≥ 16/63 (*p* = 0.021 compared to Time 1), and no anxiety was reported at 12 months in the control group (Table 3).

### Depressive symptoms

Early after the birth, depressive symptoms (BDI-II score ≥ 14/63) were more common in the interest group than in the control group (*p* = 0.003), but the prevalence was similar at 12 months (*p* = 0.733). Depressive symptoms were equally common in mothers and fathers in the interest group (Time 1: *p* = 0.398, Time 2: *p* = 1.0) and in the control group (Time 1: *p* = 1.0, Time 2: *p* = 0.343) (Table 3).

Depressive symptoms were more common when measured with the EPDS (score ≥ 10/30) than with the BDI-II (*p* < 0.001 at Time 1 and Time 2). At Time 1, when assessed with the EDPS, the prevalence of possible depression was higher in the interest group than in the control group (*p* < 0.001). In the interest group, mothers had more possible depression than fathers (*p* = 0.004). At 12 months, the prevalence of possible depression was similar between the two groups (*p* = 0.087) and between mothers and fathers (*p* = 0.736). In the interest group, the prevalence of depressive symptoms was similar at 12 months to that early after the birth (*p* = 0.296) (Table 3).

### General Stress

Moderate and high perceived stress (PSS-14 score ≥ 19/56) was common in both groups at both time points, but the groups did not differ in perceived stress at either time point (Time 1: *p* = 0.134; Time 2: *p* = 0.615). At Time 1 in the interest group, mothers had more stress than fathers (*p* = 0.001), but not at 12 months (*p* = 0.068). The prevalence of general stress was similar between the sexes in both groups; Time 1: *p* = 0.146 and *p* = 0.390, and Time 2: *p* = 0.142 and *p* = 0.254 in the interest group and the control group, respectively (Table 3).

### Posttraumatic Growth

Posttraumatic growth was measured at 12 months and in the interest group only. We did not have this data in the control group. Eight mothers out of 15 and seven fathers out of 15 had PTG (PTGI score ≥ 42/105, *p* = 0.715) (Table 3).

In the interest group, PTGI total scores correlated positively with IES-R scores (*r* = 0.388, *p* = 0.034), but correlations with other measures at 12 months were not statistically significant. The correlation coefficient was 0.041 with LS-4 scores (*p* = 0.830), 0.021 with PSS-14 (*p* = 0.912), 0.185 with EPDS (*p* = 0.328), 0.006 with BDI (*p* = 0.973), and 0.209 with BAI (*p* = 0.267).

### Traumatic Distress

Traumatic distress was measured in the interest group only, first at one month after the birth and for a second time at 12 months. The prevalence of traumatic distress (IES-R score ≥ 25/66) was similar at both time points (*p* = 0.141) and in mothers and fathers (at 1 month after the birth: *p* = 0.433, at 12 months: *p* = 0.390). Previous miscarriages (*n* = 4 mothers) did not correlate with the IES-R (at 1 month after the birth: *p* = 1.0, at 12 months: *p* = 1.0) (Table 3).

### Life Satisfaction

Life satisfaction was similar between the two groups (Time 1: *p* = 0.161; Time 2: *p* = 0.060). In the control group, all the participants were satisfied with life (LS-4 score 4–11) early after the birth compared to one dissatisfied mother and one dissatisfied father in the interest group, and at 12 months in the control group, two mothers were dissatisfied with life compared to four dissatisfied mothers and two dissatisfied fathers in the interest group. There was no difference between mothers and fathers in LS (Time 1: *p* = 1.0; Time 2: *p* = 0.260) (Table 3).

In the interest group, the LS-4 score early after the birth correlated inversely with the BAI (*r* =  − 0.612, *p* < 0.001), EPDS (*r* =  − 0.765, *p* < 0.001), BDI-II (*r* =  − 0.688, *p* < 0.001), PSS-14 (*r* =  − 0.646, *p* < 0.001), and IES-R scores (*r* =  − 0.428, *p* = 0.015). The inverse correlations were similar at 12 months: BAI (*r* =  − 0.543, *p* = 0.002), EPDS (*r* = – 0.615, *p* < 0.001), BDI-II (*r* =  − 0.773, *p* < 0.001), PSS-14 (*r* =  − 0.539, *p* = 0.003), and IES-R scores (*r* =  − 0.534, *p* = 0.002).

In the control group, the LS-4 score had a significant inverse correlation with the BAI score early after the birth (*r* =  − 0.398, *p* = 0.001), and at 12 months (*r* =  − 0.314, *p* = 0.049). There was a significant inverse correlation between the LS-4 score and the EPDS score at Time 1 (*r* =  − 0.443, *p* < 0.001) and at 12 months (*r* =  − 0.722, *p* < 0.001), and also at both time points between the LS-4 score and the BDI-II score (*r* =  − 0.425, *p* = 0.001 and *r* =  − 0.640, *p* < 0.001, respectively) and PSS-14 score (*r* =  − 0.373, *p* = 0.003, and *r* =  − 0.585, *p* = 0.001, respectively).

## Discussion

This study examined anxiety, depressive symptoms, general stress, PTG, traumatic distress, and LS among parents of seriously ill infants from the acute diagnostic period up to 12 months after their child’s initial NICU admission. The study compared the parents of seriously ill infants with those of healthy infants, and it also allowed a comparison of responses between fathers and mothers. The current study broadens our understanding of LS, PTG, and psychiatric symptoms in the parents of seriously ill infants. One of the novelties of this study was the inclusion of parents of infants treated with therapeutic hypothermia, thus providing new insight into their psychological well-being and PTG.

Severe and moderate anxiety was more common in the parents of seriously ill infants soon after the diagnosis than in those of healthy infants. Even though anxiety decreased in both groups, the parents of the ill infants were still more anxious than the control group after 12 months. This is consistent with earlier studies (e.g. Cabizuca et al., 2009; Muscara et al., 2015; Yaman & Altay, 2015). According to Kong et al. (2013), the level of social support and perceived stress are the most important factors related to parental anxiety. Psychological intervention programs individualized to the needs of parents are effective in reducing anxiety compared to standard care (Cano Giménez & Sánchez, 2015). In a single-blind randomized controlled trial, preoperative preparation was found to substantially reduce parent state anxiety (Fincher et al., 2012). The anxiety of parents of severely ill infants can be caused by concerns about the child's survival and health (Wray et al., 2011). In parents of an infant with asphyxia, it may also be due to the separation from the infant caused by therapeutic hypothermia (Laudi & Peeples, 2020). In the study of Craig et al. (2020), parents reported that the physical separation imposed by hypothermia adversely impacted on their ability to bond with their infant. In future studies, it would be important to further investigate the nature of anxiety in these parents, and to develop and evaluate interventions to alleviate it.

Depressive symptoms were common in the interest group, and they did not decrease at the 12-month follow-up. Mothers had more depressive symptoms than fathers measured with the EPDS. In the longitudinal cohort study of Bergström et al. (2012), the incidence of postpartum depression (PPD) among mothers of infants cared for in the NICU was 15% at one month and 14% at four months. Mothers who experienced PPD at 1 month had an almost eightfold increased risk of experiencing PPD at four months. Women who were not offered counseling during their infant’s stay in the NICU had a 60% increased risk of PPD onset. Mothers of infants with neonatal hypoxic–ischemic encephalopathy (HIE) were at high risk of developing PPD, which may in part be related to therapeutic hypothermia interfering with maternal–infant bonding (Laudi & Peeples, 2020). The results of our research support earlier findings that infant care in the NICU, and particularly therapeutic hypothermia, adds to the risk of maternal depression. According to our research, depressive symptoms were long-lasting, and it would therefore be important to screen parents’ needs for psychological support even after the initial phase.

**Fig. 1:**
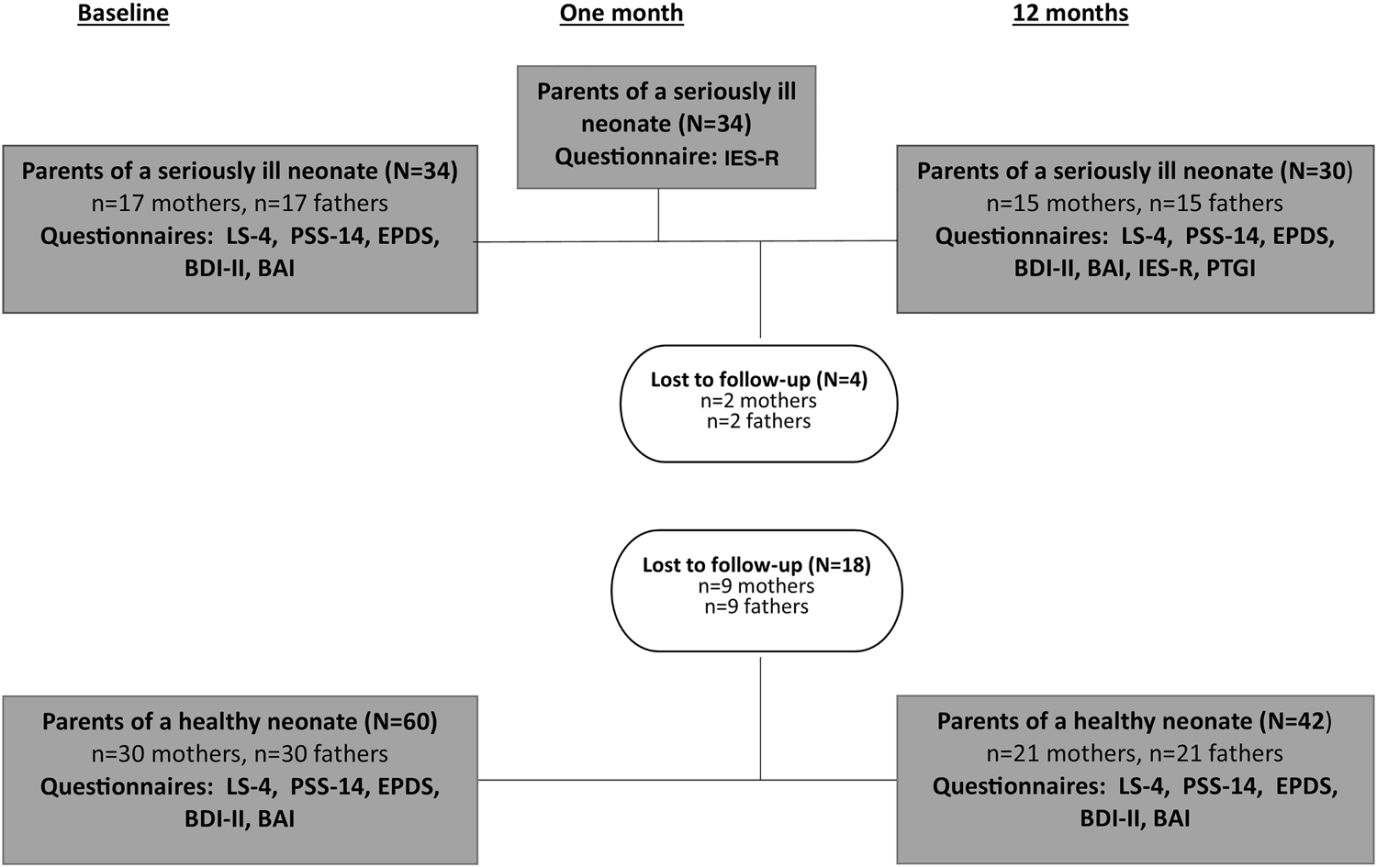
Flow chart for the study. The questionnaires were the 4-item Life Satisfaction Scale (LS-4), the Perceived Stress Scale-14 (PSS-14), the Edinburgh Postnatal Depression Scale (EPDS), the Beck Depression Inventory—Second Edition (BDI-II), the Beck Anxiety Inventory (BAI), the Impact of Event Scale–Revised (IES-R), and the Posttraumatic Growth Inventory (PTGI)

In our research, moderate or high perceived stress was common among the parents of both seriously ill and healthy infants. The perceived stress had not reduced at the 12-month follow-up in either group, which may indicate the burden of the baby year not only for parents of ill infants but also for those of healthy infants. The depressive symptoms of parents with healthy infants had increased on follow-up, so it would also be important to screen their mood and stress level during the first baby year, for example, when visiting a child health clinic. In a previous study by Enke et al. (2017), parents of a younger age and those of infants with severe prognoses were more likely to experience stress. Staff in the NICU should communicate empathetically and help to reduce stress in parents particularly at risk. Recognizing mood symptoms and stress would be relevant not only for the parents’ own but also for the child’s well-being. Azak et al. (2013) found that infants of mothers with comorbid anxiety and depression had relatively higher cortisol production from morning to bedtime and higher bedtime values than infants of non-depressed mothers (Fig. 1).

The risk of PTSD was relatively high among the parents, especially the mothers, of ill infants. During the 12-month follow-up, PTSD symptoms decreased in the fathers but not in the mothers. In previous studies (e.g. Aftyka et al., 2017a, 2017b), mothers have been found to have a higher prevalence and severity of PTSD than fathers. The present study found no difference between the parents in dichotomized results; nonetheless, the total amount of traumatic distress decreased in the fathers but not in the mothers. Thus, it would be important to screen and treat the trauma symptoms of parents of severely ill children during and after the baby year. In the future, it would be important to investigate the factors that explain the differences between parental trauma symptoms. It would be interesting to study, for example, how mothers’ experiences of giving birth to a child and the type of delivery affect their psychiatric symptoms and experiences of the child’s illness. According to Craig et al. (2020), the birth was frequently described as traumatic with descriptions of chest compressions, excessive blood loss, and infants not crying. Trauma was also described in the parental observations of the shivering hypothermic infant. Infant care in the NICU is also associated with a risk of a variety of complications that can intensify parental perceptions of vulnerability of the child and their worry about the child’s survival.

In our research, half of the parents of seriously ill infants reported PTG in the PTGI. In earlier studies, the prevalence of PTG among the parents of seriously ill children has varied between 37 and 88% (Barr, 2011; Colville & Cream, 2009; Hungerbuehler et al., 2011; Rodríguez-Rey & Alonso-Tapia, 2019). Posttraumatic growth has been higher in mothers than in fathers (Aftyka et al., 2020). Parents of children who were ventilated and parents of older children have reported statistically higher PTG than other parents. Aftyka et al. (2020) found that in the fathers, a significant predictor of PTG in stressful situations was the use of strategies aimed at seeking emotional support and positive reinterpretation and growth. The predictors of PTG in the mothers were seeking emotional support, religious coping, and planning. Aftyka et al. (2020) concluded that the parents should be provided to a greater extent with psychological and psychotherapeutic help, which would provide them with both emotional support and the possibility of a positive reinterpretation of difficult events.

Parental LS was quite high for the parents of both seriously ill and healthy infants. Life satisfaction declined in the parents of healthy infants at the 12-month follow-up, but not in the parents of seriously ill infants. Thus, despite their psychiatric symptoms and strain, the parents of seriously ill infants managed to maintain overall satisfaction with their lives. This may be related to their resilience and is consistent with previous studies (e.g. Isokääntä et al., 2019; Picoraro et al., 2014). According to Ferrand et al. (2018), parental resilience was a key factor in their envisioning good quality of life after the NICU, and less resilient parents were 10 times more likely to predict that their newborn would remain chronically ill. Parental projection of the future quality of life was not associated with the child’s risk of disability. Parental resilience was not diminished by the stress of hospitalization itself and 75% of parents had good resilience (Ferrand et al., 2018). Taken together, these findings should lead to increased awareness of the importance of LS and resilience, as well as identifying and supporting parents with lower resilience.

### Limitations and Future Research Implications

The main limitation of the present study was its small sample size. However, we were not able to recruit more dyads of seriously ill infants during the 4-year study period. Second, qualitative and mixed-methods research could shed more light on the nature of parental experiences. Longitudinal, prospective research would also capture more about the development of parental PTG and psychiatric symptoms as the child grows (Tennen, & Affleck, 2009). In the future, it would also be important to investigate the factors that make it possible for parents to maintain LS, despite their child's serious illness. For example, the importance of parenting and couple relationship satisfaction for overall LS should be explored.

At the university hospital where this research was carried out, the psychosocial support services of the NICU were available. All the parents in the interest group had the opportunity to use the services of a crisis worker, a social worker, a baby family worker, and a priest. When considered necessary, a baby family nurse made home visits and appointments with a child psychiatrist were provided. This study was limited to the quantitative data obtained through the questionnaires, but previously published qualitative studies provide important perspectives on the parental needs for support in NICUs. Nurses have an essential role in providing family centered care in NICUs; they provide individual support, educate parents, promote open communication, and encourage meaningful involvement (Gilstrap, 2021; Nassef et al., 2013). These sense giving strategies enhance the understanding of the parents and their participation in neonatal care practices. According to Segre et al. (2016), nurse-delivered depression screening and counseling could increase the detection of depression as well as treatment use among at-risk mothers. Moreover, neonatologists have a central role in facilitating parental participation in decision-making (Axelin et al., 2018). In addition, peer support has been very influential in development of the maternal role in the NICU (Rossman et al., 2015). Psychological, art-based, and musical interventions have also been effective in supporting the psychological well-being of parents in the NICU (e.g. Ettenberger et al., 2017; Mendelson et al., 2018; Mouradian et al., 2013). Support for the parents should continue after the hospitalization of the child, because they have many uncertainties after their child’s discharge from the NICU (Dellenmark & Wigert, 2014; White et al., 2017). In the future, it would be important to assess the impact of the existing supportive methods, as well as develop new interventions and investigate their effectiveness in supporting parental well-being.

### Conclusions

In this study, the parents of newborn infants receiving therapeutic hypothermia and surgical treatment in the NICU had a variety of psychiatric symptoms after the infant was diagnosed with the severe condition and a year after the diagnosis. Symptoms of depression, general stress, and traumatic distress were common and relatively persistent. Parents also had traumatic growth, and they were rather satisfied with their lives, despite their symptoms. Healthcare professionals need education and information on the psychological well-being of parents to identify those parents in need of help and guide them to the psychological support they need.
